# Supplementary material for: Unique roles of co-receptor-bound LCK in helper and cytotoxic T cells
Source: Nat Immunol. 2022 Dec 23;24(1):174–85. doi: 10.1038/s41590-022-01366-0 (PMC9810533; doi:10.1038/s41590-022-01366-0)
Supplement: Supplementary file 1 — Supplementary Figs. 1–6. [file 41590_2022_1366_MOESM1_ESM.pdf]

nature immunology

Article

https://doi.org/10.1038/s41590-022-01366-0

Unique roles of co-receptor-bound LCK in helper and cytotoxic T cells

In the format provided by the authors and unedited

Supplementary Figure 1

a Gating strategy: Thymus

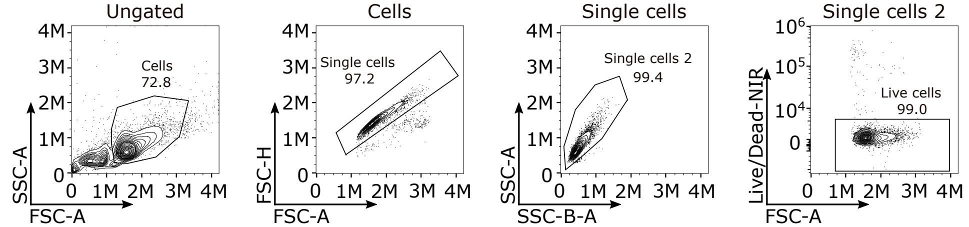

b Gating strategy: pTCRζ

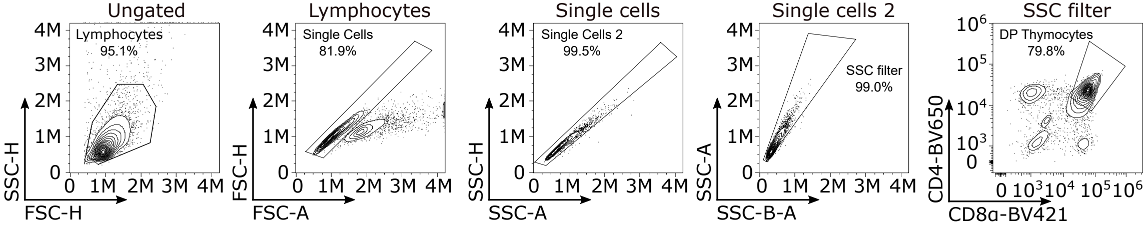

c Thymocytes: Gating strategy pZAP70 and pTCRζ

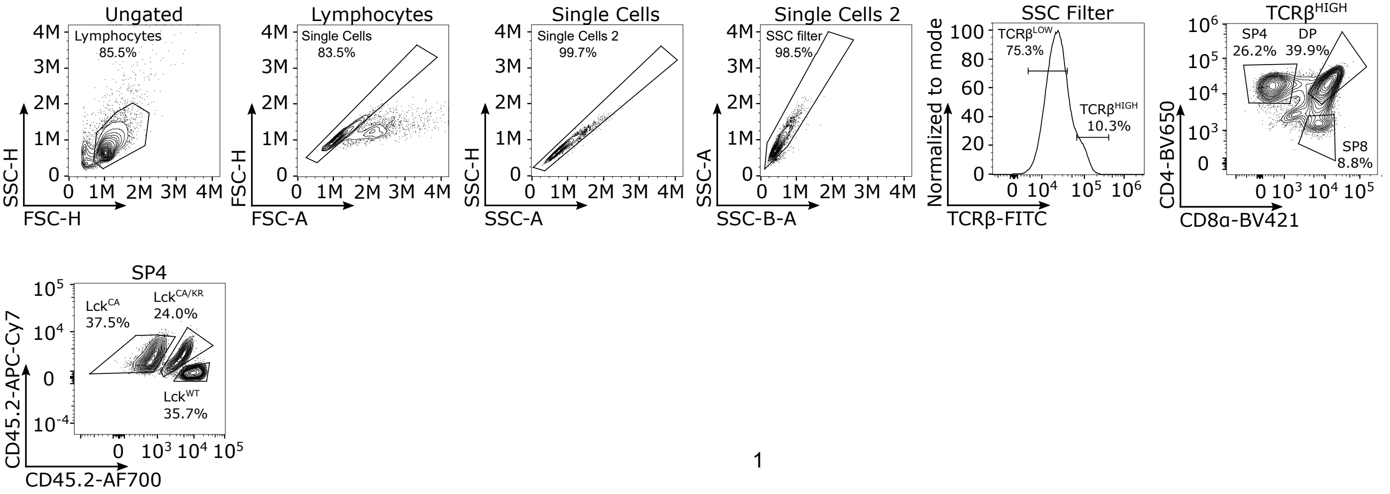

Supplementary Figure 1. Gating strategy for the flow cytometry analysis of thymocytes

(a) An example of the gating strategy used for live cell flow cytometry analysis throughout this manuscript (originating from an experiment shown in Fig.1b).

(b) An example of gating strategy for experiments in Extended Data Fig. 3e-f.

(c) An example of gating strategy from Extended Data Fig. 4e-h.

Supplementary Figure 2

a OT-I Rag2$^{KO/KO}$ Thymocytes: Gating Strategy

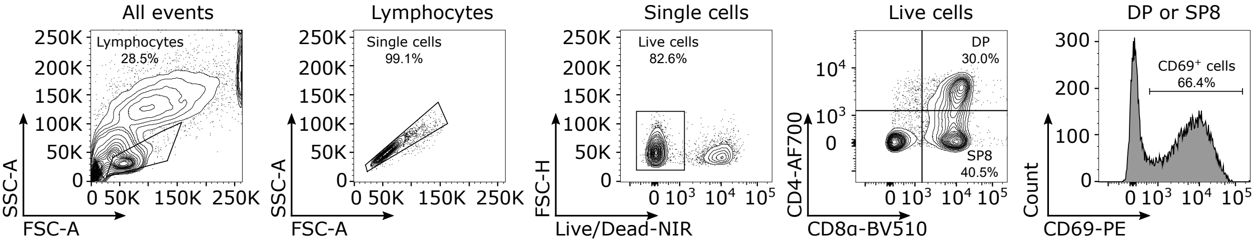

b OT-I Rag$^{KO/KO}$ β2m$^{KO/KO}$ FTOC Gating strategy

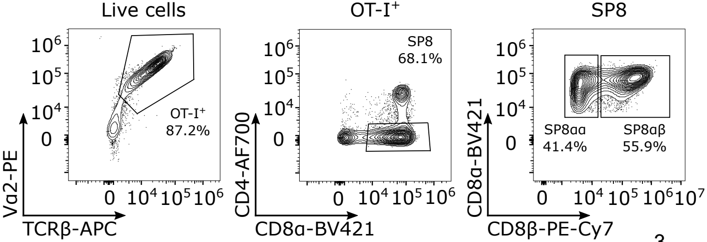

Supplementary Figure 2. Gating strategy for the analysis of OT-I thymocyte activation and fetal thymic organ cultures.

(a) An example of gating strategy for Fig.2c.

(b) An example of gating strategy for fetal thymic organ cultures (FTOC) from Extended Data Fig. 5b.

Supplementary Figure 3

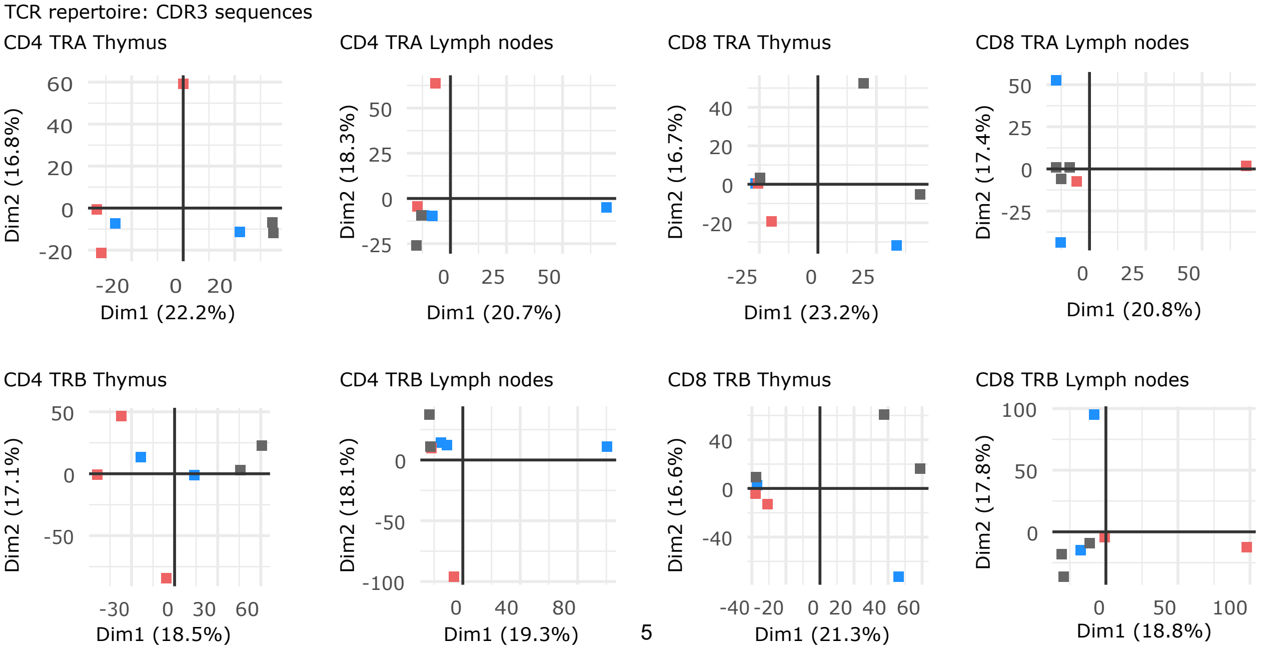

Supplementary Figure 3. Principal component analysis of TCRα (TRA) and TCRβ (TRB) CDR3 amino acid sequences.

The principal component analyses of the normalized counts of the amino acid CDR3 sequences of the TCRα (TRA) and TCRβ (TRB) chains in CD4 and CD8 LNs T cells and mature SP4 and SP8 thymocytes from $Lck^{WT/WT}$ (gray), $Lck^{CA/CA}$ (blue) and $Lck^{CA/KR}$ (red) mice. TRAV11-TRAJ18 sequences and sequences occurring in less than five CD4 or in less than five CD8 samples were removed before the normalization. n = 2 or 3 replicates from independent experiments per group (Supplementary Table 4). Related to Fig. 2e-g.

Supplementary Figure 4

Top 20 most abundant CDR3 sequences

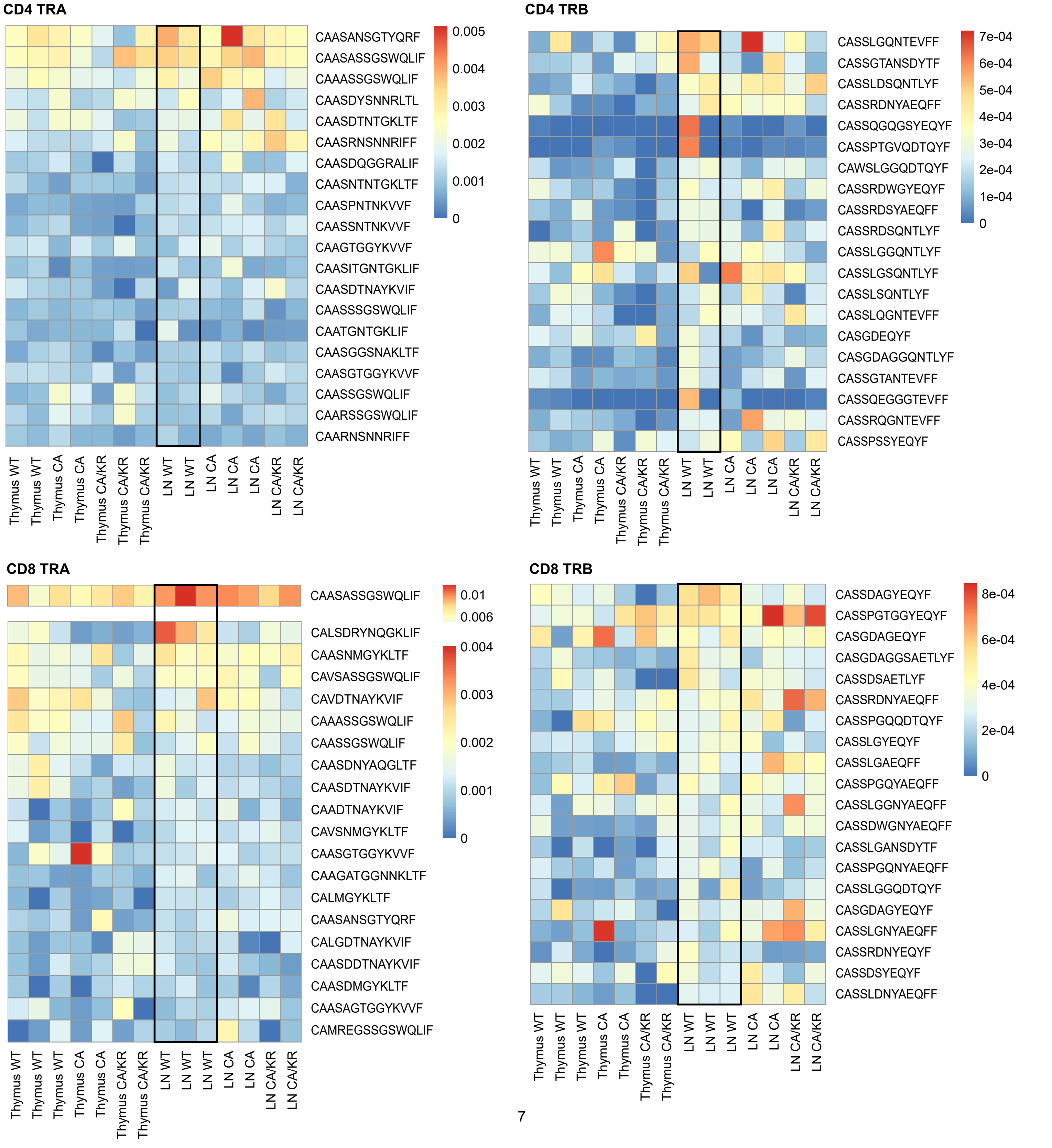

Supplementary Figure 4. Frequencies of the top 20 most abundant clonotypes from *Lck*$^{WT/WT}$ mice.

Heat maps show the relative frequency of the top 20 most abundant TCRα (TRA) or TCRβ (TRB) CDR3 sequences from *Lck*$^{WT/WT}$ mice in CD4 and CD8 LNs T cells and mature SP4 and SP8 thymocytes from indicated *Lck* variant mice. TRAV11-TRAJ18 sequences were removed before the normalization. Related to Fig. 2e-g.

Supplementary Figure 5

Repertoire overlap (unique CDR3 sequences)

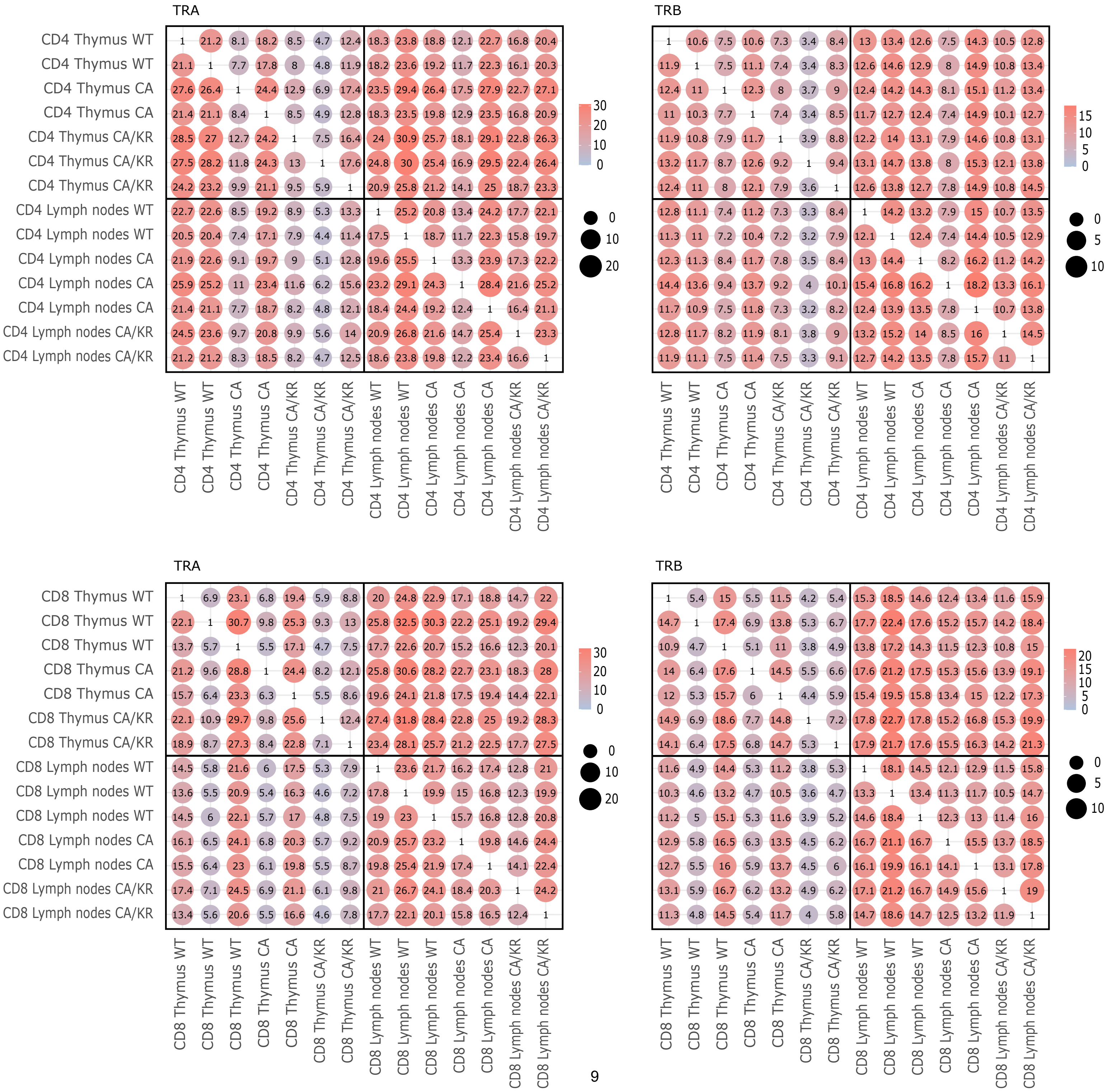

Supplementary Figure 5. Overlap of TCR repertoires of *Lck*$^{WT/WT}$, *Lck*$^{CA/CA}$, and *Lck*$^{CA/KR}$ mice.

The repertoire overlap was calculated as the percentage of the unique CDR3 amino acid sequences from the sample in the row present among the unique CDR3 sequences in each of the other samples (in columns). Related to Fig. 2e-g.

Supplementary Figure 6

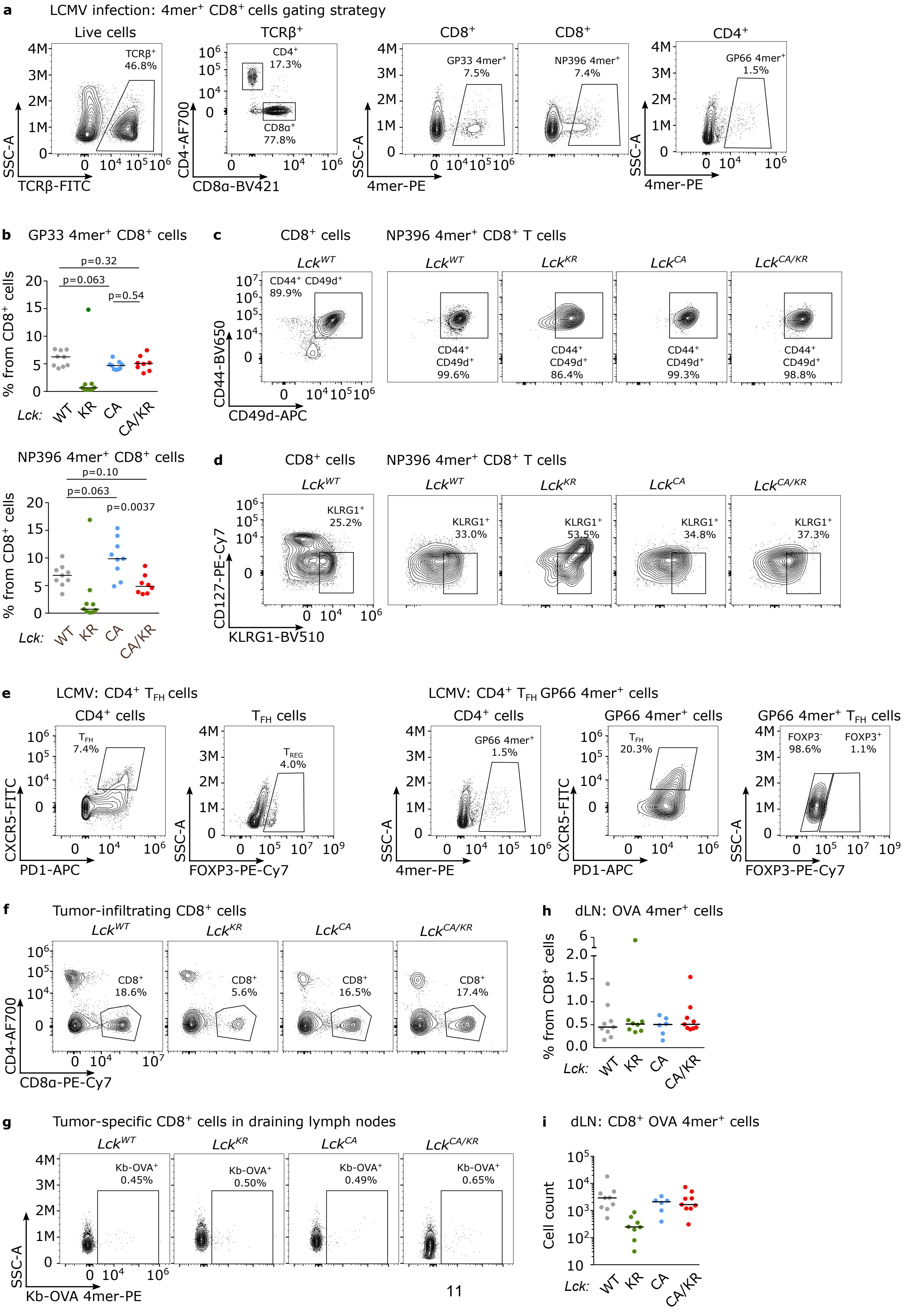

Supplementary Figure 6. Analysis of the anti-viral T cell response in the *Lck* variant mice.

(a-e) The indicated *Lck* variant mice were infected with LCMV and the splenocytes were analyzed on day 8 post-infection. n=8 ($Lck^{CA/KR}$) or 9 (other strains) mice in 2 ($Lck^{CA/CA}$) or 3 (other strains) independent experiments. The same experiments as shown in Extended Data Figure 7a-g.

(a) An example of gating strategy for the detection of LCMV-specific T cells using tetramers.

(b) Quantification of the frequencies of D^b-GP33 4mer$^+$ and D^b-NP396 4mer$^+$ T cells among all CD8$^+$ T cells. The statistical analysis was calculated using Mann-Whitney test.

(c) A representative experiment showing the percentage of CD44$^+$ CD49d$^+$ activated cells among D^b-NP396 4mer$^+$ CD8$^+$ T cells in the indicated mice (Extended Data Fig. 7d).

(d) A representative experiment showing the percentage of short-lived effector KLRG1$^+$ CD127$^-$ cells among D^b-NP396 4mer$^+$ CD8$^+$ T cells in the indicated mice (Extended Data Fig. 7e).

(e) An example of the gating strategy for the identification of CXCR5$^+$ PD-1$^+$ T_{FH} cells among CD4$^+$ T cells, FOXP3$^+$ regulatory T cells among T_{FH} cells, I-Ab-GP33 4mer$^+$ cells among CD4$^+$ T cells, CXCR5$^+$ PD-1$^+$ T_{FH} cells among I-Ab-GP33 4mer$^+$ CD4$^+$ T cells, and FOXP3$^+$ regulatory T cells among T_{FH} I-Ab-GP33 4mer$^+$ CD4$^+$ T cells (Fig. 3d-e, Extended Data Fig. 7f).

(f-i) Tumor-infiltrating CD8$^+$ T cells were isolated from MC-38-OVA tumors from the indicated *Lck* variant mice. n=9 for $Lck^{WT/WT}$, $Lck^{CA/KR}$, n=8 for $Lck^{CA/CA}$, $Lck^{KR/KR}$ from 3 independent experiments. The same experiments as shown in Extended Data Fig. 7h-j.

(f) An example of the gating strategy for the identification of CD8$^+$ T cells in the tumor (gated on viable lymphocytes) from the indicated *Lck* variant mice.

(g) An example of the gating strategy for the identification of K^b-OVA 4mer$^+$ in the tumor-draining LNs (gated on viable CD8$^+$ lymphocytes) from the indicated Lck variant mice.

(h-i) Quantification of the frequency (h) and counts (i) of K^b-OVA 4mer$^+$ CD8$^+$ T cells in the tumor-draining LNs in indicated *Lck* variant mice. Individual mice and medians are shown

Supplementary Figure 7

a Gating strategy: OT-I CD8$^+$ cells

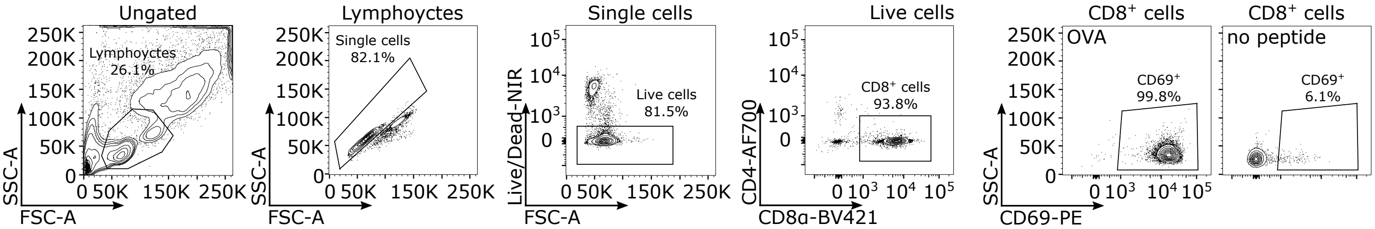

b Gating strategy: Jurkat cells

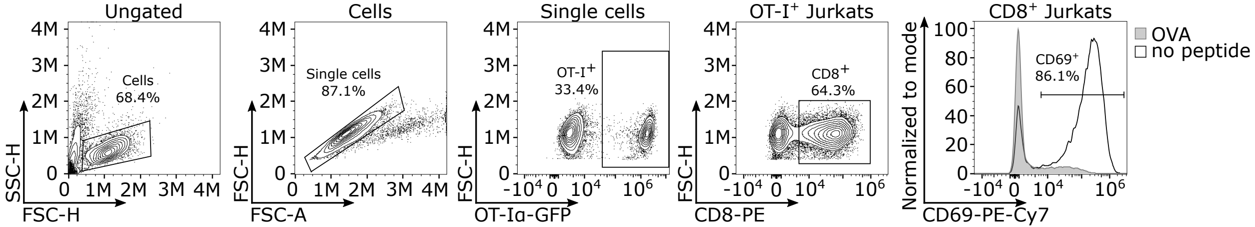

Supplementary Figure 7. Gating strategy for OT-I peripheral T cells and OT-I Jurkat cells.

(e) An example of gating strategy for the evaluation of *ex vivo* activation of primary T-cells (Fig. 4e).

(h) An example of gating strategy for the evaluation of activation of OT-I Jurkat T cells by OVA-loaded T2-Kb cells (Fig. 4f).
