# Supplementary material for: Unique roles of co-receptor-bound LCK in helper and cytotoxic T cells
Source: Nat Immunol. 2022 Dec 23;24(1):174–85. doi: 10.1038/s41590-022-01366-0 (PMC9810533; doi:10.1038/s41590-022-01366-0)
Supplement: Supplementary file 2 — Reporting Summary [file 41590_2022_1366_MOESM2_ESM.pdf]

nature portfolio

Corresponding author(s): Ondrej Stepanek

Last updated by author(s): Oct 17, 2022

Reporting Summary

Nature Portfolio wishes to improve the reproducibility of the work that we publish. This form provides structure for consistency and transparency in reporting. For further information on Nature Portfolio policies, see our Editorial Policies and the Editorial Policy Checklist.

Statistics

For all statistical analyses, confirm that the following items are present in the figure legend, table legend, main text, or Methods section.

n/a	Confirmed	
☐	☒	The exact sample size (*n*) for each experimental group/condition, given as a discrete number and unit of measurement
☐	☒	A statement on whether measurements were taken from distinct samples or whether the same sample was measured repeatedly
☐	☒	The statistical test(s) used AND whether they are one- or two-sided<br>*Only common tests should be described solely by name; describe more complex techniques in the Methods section.*
☒	☐	A description of all covariates tested
☐	☒	A description of any assumptions or corrections, such as tests of normality and adjustment for multiple comparisons
☐	☒	A full description of the statistical parameters including central tendency (e.g. means) or other basic estimates (e.g. regression coefficient) AND variation (e.g. standard deviation) or associated estimates of uncertainty (e.g. confidence intervals)
☐	☒	For null hypothesis testing, the test statistic (e.g. *F*, *t*, *r*) with confidence intervals, effect sizes, degrees of freedom and *P* value noted<br>*Give P values as exact values whenever suitable.*
☒	☐	For Bayesian analysis, information on the choice of priors and Markov chain Monte Carlo settings
☒	☐	For hierarchical and complex designs, identification of the appropriate level for tests and full reporting of outcomes
☒	☐	Estimates of effect sizes (e.g. Cohen's *d*, Pearson's *r*), indicating how they were calculated

Our web collection on statistics for biologists contains articles on many of the points above.

Software and code

Policy information about availability of computer code

Data collection	MetaMorph imaging software (MM 7.10.1.161, Molecular Devices) was used for the collection of microscopy images for FRET measurements.
Data analysis	GraphPad Prism for Windows Version 5.04, FlowJo 10.6.2 with EmbedSOM plugin, ImageJ 1.53c, Pattern (pattern.img.cas.cz), R (version 4.2.1) including the packages immunarch (v0.6.9), pheatmap (v1.0.12) factoextra (v1.0.7) and tidyverse (v1.3.1), MiXCR (v3.0.13)

For manuscripts utilizing custom algorithms or software that are central to the research but not yet described in published literature, software must be made available to editors and reviewers. We strongly encourage code deposition in a community repository (e.g. GitHub). See the Nature Portfolio guidelines for submitting code & software for further information.

Data

Policy information about availability of data

All manuscripts must include a data availability statement. This statement should provide the following information, where applicable:

- Accession codes, unique identifiers, or web links for publicly available datasets
- A description of any restrictions on data availability
- For clinical datasets or third party data, please ensure that the statement adheres to our policy

Data of the TCR repertoire analysis are available in the Sequence Read Archive (PRJNA872031). Raw flow cytometry data and microscopy images are available upon a reasonable request to the corresponding author. All other data generated or analyzed during this study are included in this published article (and its supplementary information files).

Field-specific reporting

Please select the one below that is the best fit for your research. If you are not sure, read the appropriate sections before making your selection.

☒ Life sciences ☐ Behavioural & social sciences ☐ Ecological, evolutionary & environmental sciences

For a reference copy of the document with all sections, see nature.com/documents/nr-reporting-summary-flat.pdf

Life sciences study design

All studies must disclose on these points even when the disclosure is negative.

Sample size	No sample size calculation was performed. In animal experiments, we usually aimed at the minimal number of 10 mice per group in at least 3 independent experiments, which was based on our prior experience. The actual number of mice and experiments (usually higher than the minimal numbers) was given by the availability of mice with particular genotypes, which could be not be predicted in advance as we largely used littermates for experiments. For ex vivo experiments with primary cells and cell lines, at least 2-3 independent experiments were performed, which is the minimal number to assess the reproducibility.
Data exclusions	1. Very rare flow cytometry samples with apparent technical issues, such as bubbles in the flow cell or clogged machine, were identified based on abnormal FSC vs. SSC profile and excluded before the analysis. This is a common practice and thus, this is a pre-established criterium.<br>2. At one point, new stocks of Listeria were wrong. The reason was unknown, but the probable cause was little experience of the person, who prepared them for the first time. This was realized upon the CFU number counting. We calculate the CFU concentration from OD values and verify it by plating the Listeria suspension on plates and manual colony counting on the next day. In this case, there was almost ten times fewer colonies than expected and the experiment was excluded. The whole respective experiments were excluded. This was a pre-established criterium.<br>3. In one experiment focused on LCMV titers, one sample was removed because of bad RNA quality. The reference host gene was amplified at much later cycles than usual (i.e., more than 4 cycles). This was not an explicitly pre-established criterium, but we decided to exclude this sample that was apparently wrong from the technical point of view.<br>4. We originally aimed to have 3 samples (3 independent experiments) per group for the TCR sequencing experiment. Unfortunately, several of the libraries failed the QC on Agilent and were not included in the sequencing run, resulting in only 2 samples in some groups.
Replication	All data were replicated in at least 2, but usually 3 or more, independent experiments (this is indicated in the Figure Legend). The aggregate data are shown in the paper. There were no unsuccessful attempts to replicate the experimental results.
Randomization	The mice and transgenic cells lines were allocated to experimental groups solely based on their genotype. If more experimental conditions were used in a single experiments (such as different Listeria strains), the allocation of the mice were random (i.e., based on mouse ID in the database, before the experimenter had any contact with them) in the way that or sex- and age-matched animals with different genotypes were compared (preferably littermates). For the cell line transfection/transduction experiments, identical cell culture aliquots of the split parental culture were used, thus no randomization was required.
Blinding	The allocation of the mice was based solely on their genotype. The experimenter processed the mice based on their ID number (i.e., without the information about the genotype). ID was matched with the genotype only during the data analysis at the end of the experiment. Because no subjective scoring method was used, the analysis of the mice was not explicitly blinded. Ex vivo experiments with primary cells and cell lines were not blinded. Because no subjective scoring method was used for the analysis, the blinding was not necessary.

Reporting for specific materials, systems and methods

We require information from authors about some types of materials, experimental systems and methods used in many studies. Here, indicate whether each material, system or method listed is relevant to your study. If you are not sure if a list item applies to your research, read the appropriate section before selecting a response.

Materials & experimental systems

n/a	Involved in the study
☐	☒ Antibodies
☐	☒ Eukaryotic cell lines
☒	☐ Palaeontology and archaeology
☐	☒ Animals and other organisms
☒	☐ Human research participants
☒	☐ Clinical data
☒	☐ Dual use research of concern

Methods

n/a	Involved in the study
☒	☐ ChIP-seq
☐	☒ Flow cytometry
☒	☐ MRI-based neuroimaging

Antibodies

Antibodies used	For the analysis of murine thymocytes and T cells, the following antibodies were used: anti-CD4 (clone RM4-5, BioLegend #100536, #100545, #130310, diluted 200× and RM4-4, BioLegend #116004, diluted 200x), anti-CD8α (clone 53-6.7, BioLegend #100738,

#100753, #100708, #100722, diluted 200x), anti-CD8β (clone YTS156.7.7, BioLegend #126615, diluted 200×), anti-CD24 (clone M1/69, BioLegend #101806, diluted 200×), anti-CD25 (clone PC61, BioLegend #102016 diluted 400x, #102006 and #102036, diluted 200x), anti-CD44 (clone IM7, BioLegend #103049, diluted 200×), anti-CD45.1 (clone A20, BioLegend #110723, diluted 200×), anti-CD45.2 (clone 104, BioLegend #109808, diluted 200×), anti-CD49d (clone R1-2, BioLegend #103618, #103622, diluted 200×), anti-CD69 (clone H1.2F3, BioLegend #104508, diluted 200×), anti-TCRβ (clone H57-597, BioLegend #109218, #109206, #109243, #109212, BD Pharmingen #553171, diluted 200-400x), anti-PD-1 (clone 29F.1A12, BioLegend #135209, diluted 200x), anti-CXCR5 (clone L138D7, BioLegend #145504, #145520, diluted 200x), anti-KLRG1 (clone 2F1/KLRG1, BioLegend #138421, #138410, diluted 200x), anti-FOXP3 (clone FJK-16s, eBioscience #25-5773-82, diluted 100x), anti-CD127 (clone A7R34, BioLegend #135013, diluted 200x). For analysis of Jurkat cell lines anti-CD4 (clone MEM-241, Exbio #A7-359-T100, diluted 50×), anti-CD8 (clone MEM-31, Exbio #1P-207-T025, diluted 50×), anti-CD69 (clone FN50, Exbio #T7-552-T100, diluted 100×), anti-CD271 (clone ME20.4, BioLegend #345108, #345106, diluted 200×) antibodies were used. Antibodies were conjugated with various fluorophores by the manufacturers.
For basal signaling analysis, the cells were stained with anti-Phospho-ZAP-70/SYK Y319 (polyclonal, Cell Signaling #2701, diluted 30×) and pTCRζ-PE (K25-407.69, BD Biosciences #558448, diluted 20x) antibodies overnight at 4°C protected from light and then with antibodies for surface markers and with goat-anti-rabbit-Alexa Fluor555 antibody (polyclonal, Thermo Fisher Scientific #A-32732, lot:1858260, 1000x diluted) in the case of pZAP70 staining
For surface CD4 and CD8 immunoprecipitation, 2-3×10e7 of live cells were stained with biotinylated anti-CD8β (clone 53-5.8, BioLegend, #140406, 2 μg) or anti-CD4 (clone H129.19, BD, #553649, 2 μg) antibodies.
For immunoblotting, following antibodies were used: murine anti-LCK (3A5, Santa Cruz, #sc-433, diluted 200x) and rabbit mAb anti-CD8α (D4W2Z, Cell Signaling, diluted 1000x) or anti-CD4 (D7D2Z, Cell Signaling, diluted 1000x), anti-LCK (3A5, Santa Cruz, #sc-433, diluted 200x), rabbit anti-β actin (#4967, Cell Signaling, diluted 1000x) and rabbit polyclonal anti-LAT antiserum, anti-CD3-ζ (clone 6B10.2, Santa Cruz #sc-1239, diluted 50x), LCK (clone 3A5, Santa Cruz #sc-433, diluted 200-500x), anti-TCRζ (pY142) (clone K25-407.69, BD Biosciences #558402, diluted 100x), anti-ZAP70 (clone 99F2, Cell Signaling #2705S, diluted 500x), phospho-Zap-70 (Try319)/Syk (Tyr352) (Cell Signaling #2701S, diluted 50x), anti-Actin (Cell Signaling #4967, diluted 5000x), anti-pTyr (clone 4G10, Sigma Aldrich # 05-321, diluted 5000x), and anti-FLAG (clone M2, Sigma-Aldrich # F1804-200UG, diluted 1000x).

Validation

Commercially available antibodies were used in this study and we believe the trustful manufacturers performed a proper validation and QC control. We never experienced any sign that any antibody does not work properly during our work. We encourage anyone interested to check the manufacturers' web sites for specific information on validation.
The only non-commercial antibody was the anti-LAT serum. This was validated by the MW of the stained band (Extended Data Fig. 1c and the respective Source data).

Eukaryotic cell lines

Policy information about cell lines

Cell line source(s)

No cell lines available at repositories or commercial vendors were used. Jurkat LCK KO cells, and Jurkat LCK KO cells reconstituted with LCK-Flag and/or OT-I TCR GFP were generated in the research groups of some of the authors previously and were described in Courtney, A.H., et al. Mol Cell, 2017. 67(3): p. 498-511.e6 and Lo, W.L., et al. Nature Immunology, 2018. 19(7): p. 733-741, respectively.
HEK293 cells were kindly provided by Dr. Tomas Brdicka (Institute of Molecular Genetics of the Czech Academy of Sciences, Czechia).
MC38 cells were kindly provided by Prof. Ed Palmer (Department of Biomedicine, University Hospital of Basel, Switzerland).
T2-Kb were kindly provided by Prof. Ed Palmer (Department of Biomedicine, University Hospital of Basel, Switzerland).

Authentication

The identity of the Jurkat lines were confirmed by the LCK-deficiency or expression of GFP, CD8, and OT-I TCR, respectively.
The identity of MC38 and HEK293 was only based on their known morphology and adhesion to the tissue culture plastic.

Mycoplasma contamination

All cell lines were negative for mycoplasma as revealed by regular PCR testing.

Commonly misidentified lines
(See ICLAC register)

HEK293 cells are listed in the Register of cell lines that are known to be misidentified through cross-contamination or other mechanisms (iclac.org/databases/cross-contaminations/), because there was a case of their confusion with HeLa cells. We can exclude such a misidentification in our culture based on the morphology of the cells and their adhesion on the tissue culture plastic (HeLa cells need to be trypsinized), which are clearly distinct between these two lines and which were checked in each experiment. The reason for using HEK293 cells was that they are easily transfectable and negative for ZAP70, LCK, and TCRzeta, as confirmed by Immunoblotting (Extended Data Figure 2a-b).

Animals and other organisms

Policy information about studies involving animals; ARRIVE guidelines recommended for reporting animal research

Laboratory animals

The mice used in experiments had C57BL/6J background (Charles River). For the isolation of thymi for immunoblotting, 4-8 weeks old mice were used. For fetal thymic organ cultures, embryos of embryonic age E15.5 were used. In other experiments, 6-12 weeks old mice were used. Both males and females were used for experiments. We aimed at constant males and female representation among experimental groups in all experiments. The used congenic/transgenic strains were: Ly5.1 1, Cd3εKO/KO 2, OT-I Rag2KO/KO 3, 4, B3K508 Rag2KO/KO 5, Lck KO/KO 6, CD8.4 OT-I Rag2KO/KO 7,8. The colonies of all transgenic strains were established de novo in our animal facility by rederivation using embryotransfer or in vitro fertilization.

LckC20.23A/C20.23A and LckK273R/K273R knock-in mice and LckKO/KO mice were generated in the Czech Centre for Phenogenomics, IMG using 3-5 weeks old females and 9-35 weeks old males of C57BL/6N strain as parents. The founders were back-crossed on C57BL/6J background for at least 5 generations.

Mice were fed with an irradiated standard rodent breeding diet and given reverse osmosis filtered water ad libitum. They were kept in a facility with a 12h/12h light/dark cycle and temperature and relative humidity maintained at 22 ± 1 °C and 55 ± 5 %, respectively.

Wild animals	The study did not include wild animals.
Field-collected samples	The study did not include field collected samples.
Ethics oversight	Animal protocols (ID 11/2016, 115/2016, 72/2017, AVCR 2378/2022 SOVII) were approved by the Resort Professional Commission for Approval of Projects of Experiments on Animals of the Czech Academy of Sciences, Czech Republic.

Note that full information on the approval of the study protocol must also be provided in the manuscript.

Flow Cytometry

Plots

Confirm that:

☒ The axis labels state the marker and fluorochrome used (e.g. CD4-FITC).

☒ The axis scales are clearly visible. Include numbers along axes only for bottom left plot of group (a 'group' is an analysis of identical markers).

☒ All plots are contour plots with outliers or pseudocolor plots.

☒ A numerical value for number of cells or percentage (with statistics) is provided.

Methodology

Sample preparation	Single cell suspensions from the spleen, lymph nodes, and thymus were prepared by gentle meshing the organs with syringe plungers. The samples from MC38 tumors were prepared as follows: Tumors were excised from mice, cut into small pieces and incubated with 100 µg/ml Liberase (Roche #5401020001) and 50 µg/ml DNAse I (Roche #101104159001) in wash buffer (1% BSA, 1mM EDTA in HBSS w/o Ca2+/Mg2+) at 37 °C and 350 rpm shaking for 45 minutes. Mixture was resuspended with a 1000 µl wide bore pipette tip every 10 min. Undigested debris was removed by filtering through a 100 µm strainer. Cells were harvested by centrifugation at 350g at 4° C for 5 min. Pellets were resuspended in 10 ml of 40% Percoll (Cytiva #17089101) in DMEM. 10 ml of 80% Percoll in DMEM was carefully laid to the bottom of the tube to create a gradient. Samples were centrifuged at 320g at ~21 °C for 23 min with minimal ascending/descending rates. Lymphocytes present at the interphase were collected, centrifuged at 400g at 4 °C for 5 min and processed for flow cytometry analysis.
Instrument	Aurora (Cytek™ Biosciences), LSRII and FACSymphony (BD Biosciences)
Software	FlowJo 10.6.2 (BD Biosciences)
Cell population abundance	We did not sort cells in this project.
Gating strategy	The lymphocytes were gated using FSC vs SSC. The singlets were gated based on the area vs height of FSC. Viable cells were gated based on the exclusion of near-infrared LIVE/DEAD Near-IR viability dye (ThermoFisher). In the next step, cell subsets were gated based on the antibody signal, as shown in the Figures and Supplemental Figures.

☒ Tick this box to confirm that a figure exemplifying the gating strategy is provided in the Supplementary Information.
