# Supplementary figures and images for: Unique roles of co-receptor-bound LCK in helper and cytotoxic T cells
Source: Nat Immunol. 2022 Dec 23;24(1):174–85. doi: 10.1038/s41590-022-01366-0 (PMC9810533; doi:10.1038/s41590-022-01366-0)

Source Data Figure 7

LckWT

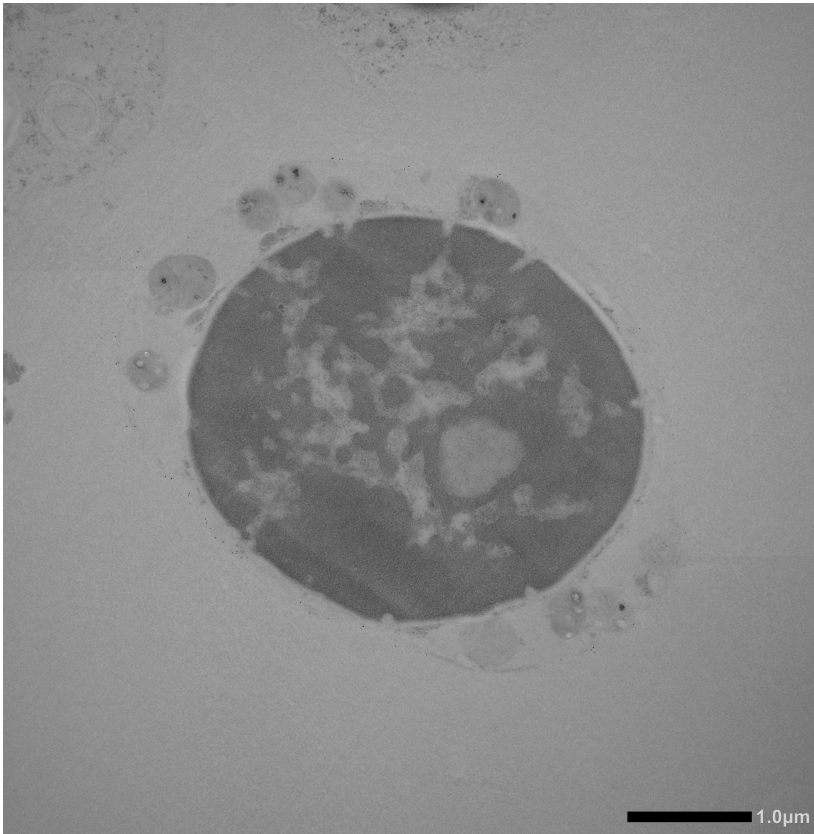

LckCA

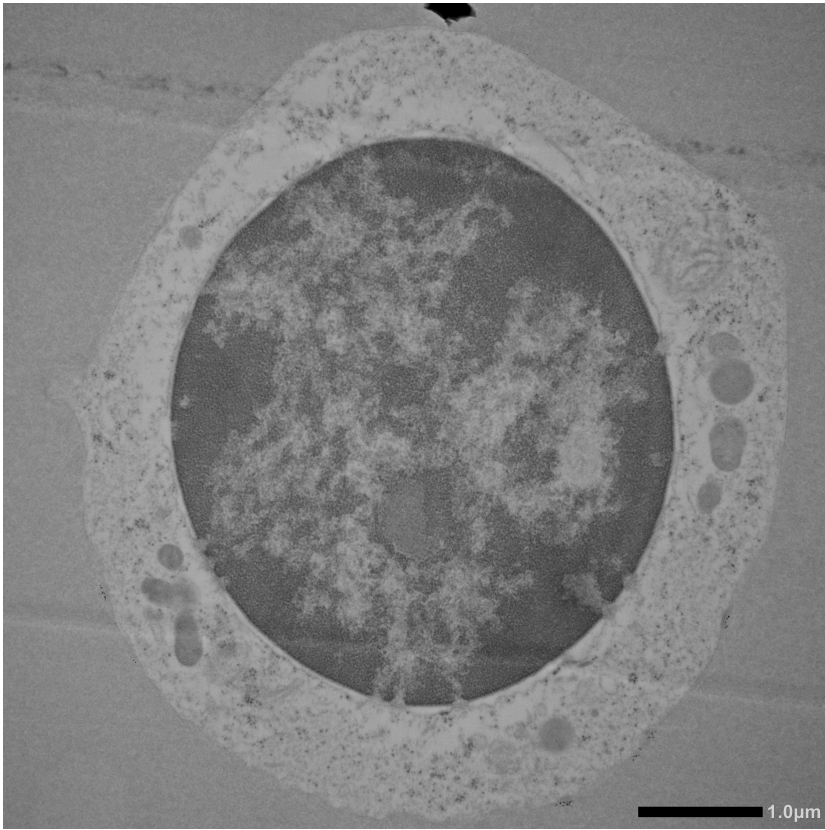

Supplement: Source Data Fig. 7 — Uncropped images. [file 41590_2022_1366_MOESM12_ESM.pdf]

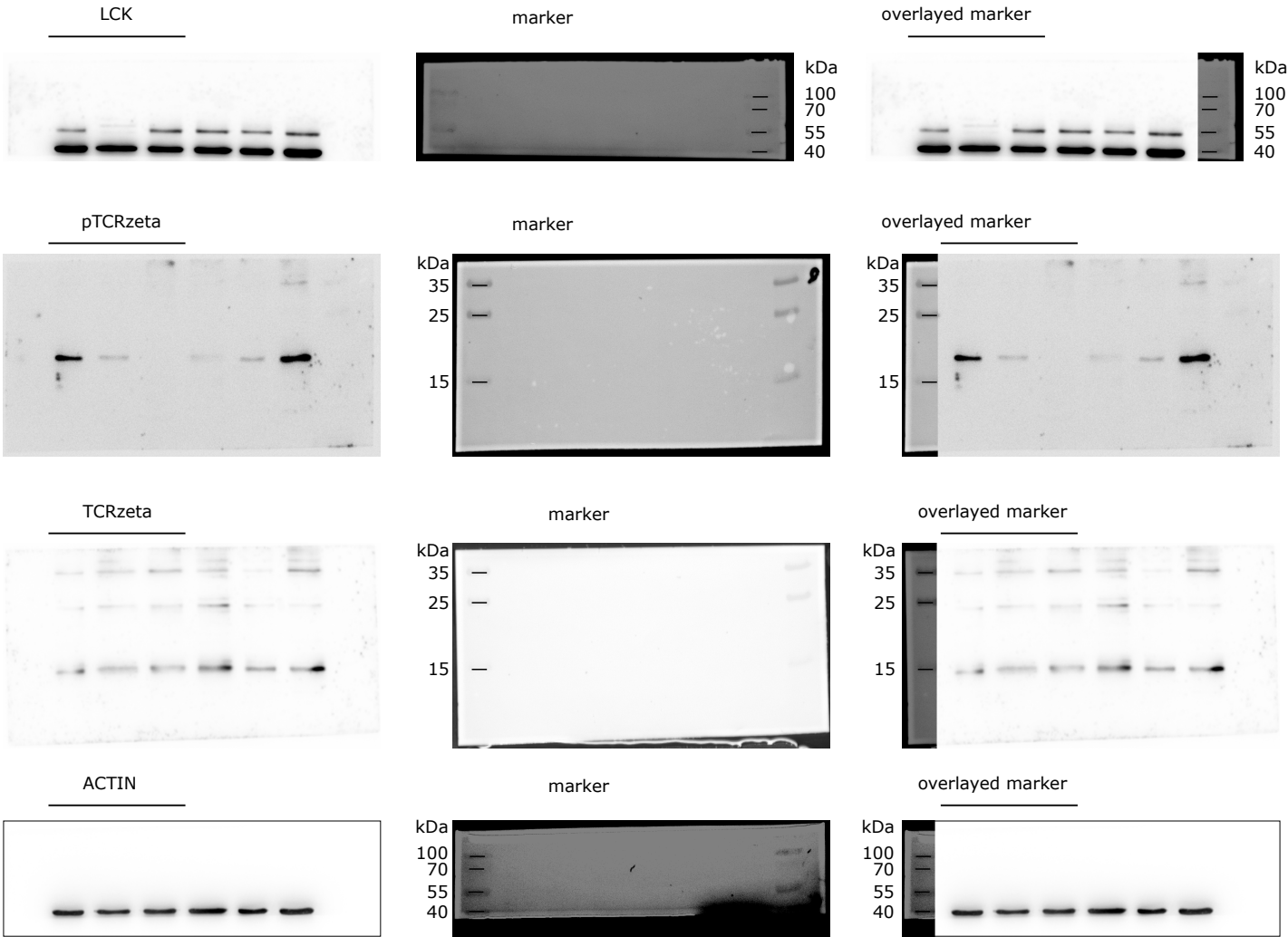

Supplement: Source Data Extended Data Fig. 3 — Uncropped immunoblots. [file 41590_2022_1366_MOESM17_ESM.pdf]
