# Supplementary material for: Unique roles of co-receptor-bound LCK in helper and cytotoxic T cells
Source: Nat Immunol. 2022 Dec 23;24(1):174–85. doi: 10.1038/s41590-022-01366-0 (PMC9810533; doi:10.1038/s41590-022-01366-0)

ExtData1c

THYMUS: Loading: WT, CA, CA/KR, KR, KO

LCK

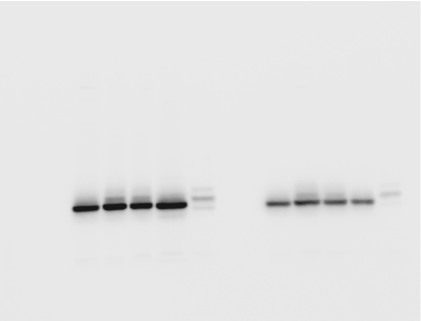

marker

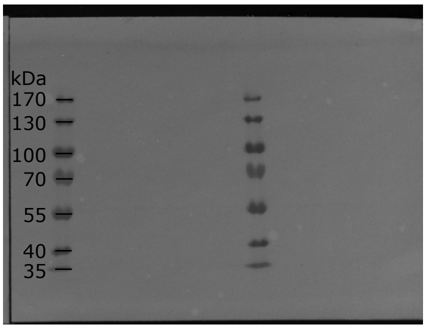

overlayed marker

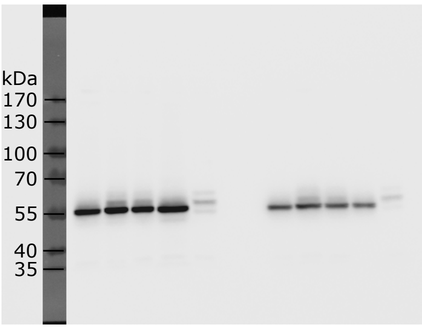

ACTIN

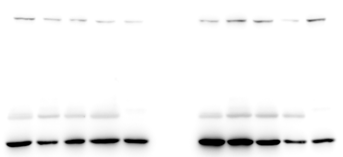

marker

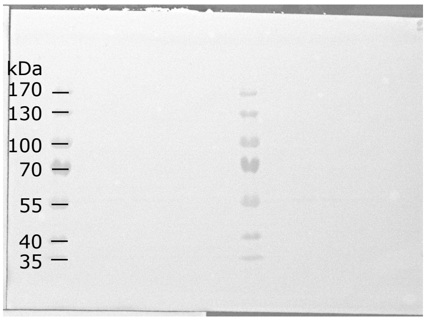

overlayed marker

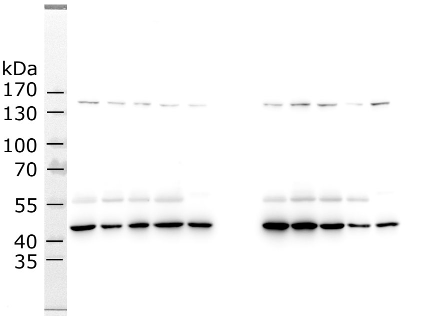

Lymph Nodes: Loading: WT, CA, CA/KR, KO, KR

LCK

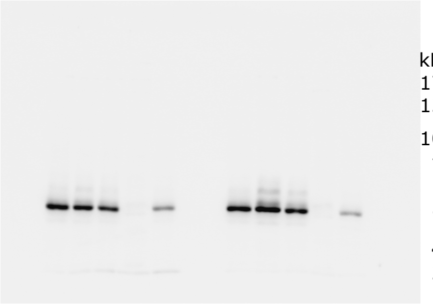

marker

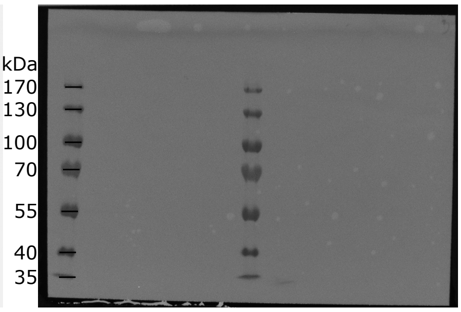

overlayed marker

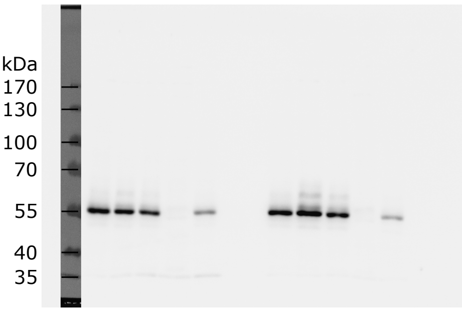

ACTIN

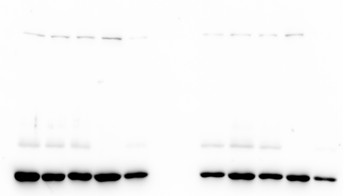

marker

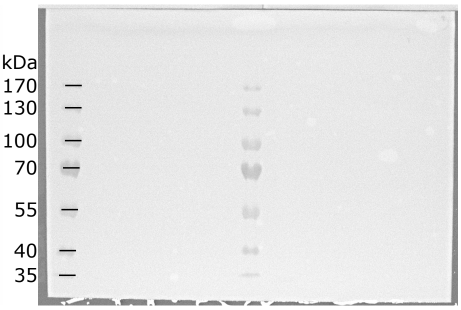

overlayed marker

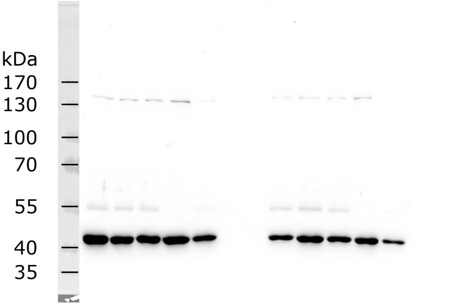

LAT

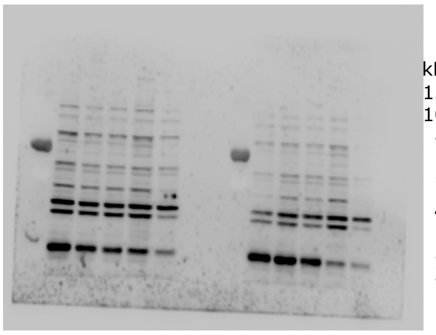

marker

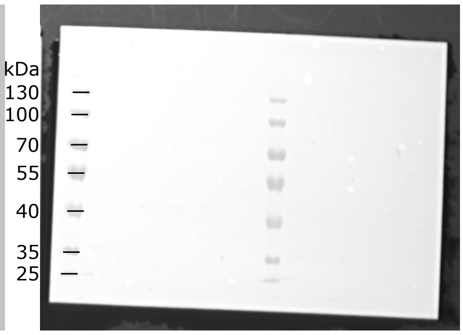

overlayed marker

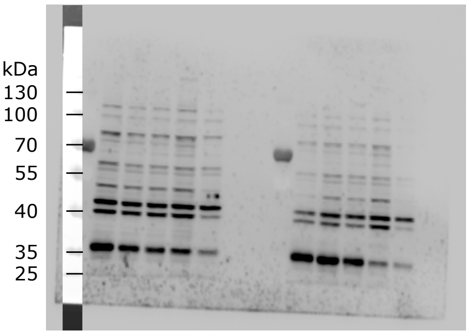

ExtData1d

THYMUS CD4-IP: Loading: WT, CA, CA/KR, KR, KO

CD4

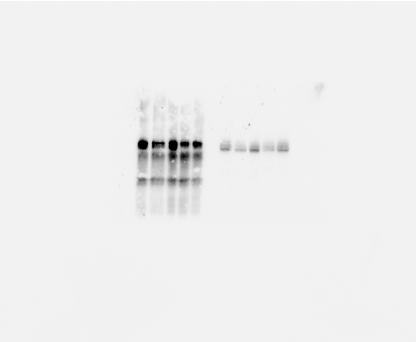

marker

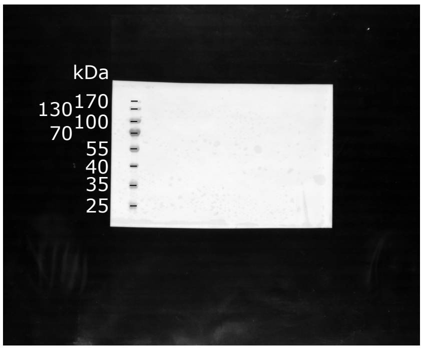

overlayed marker

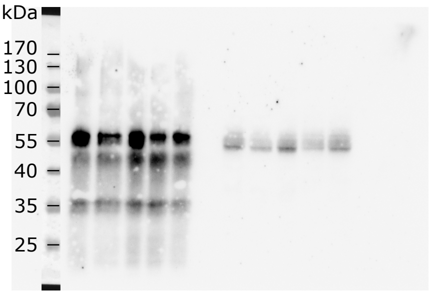

LCK

marker

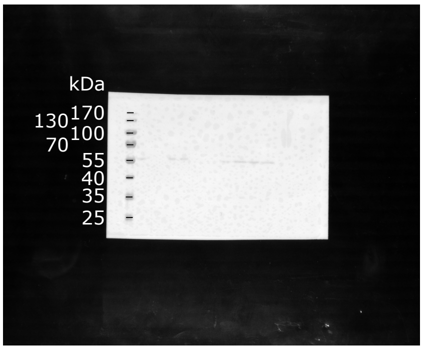

overlayed marker

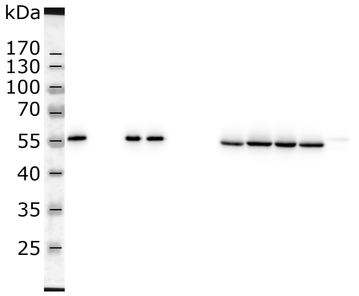

ExtData1e

THYMUS CD8b-IP: Loading: WT, CA, CA/KR, KR, KO

CD8a

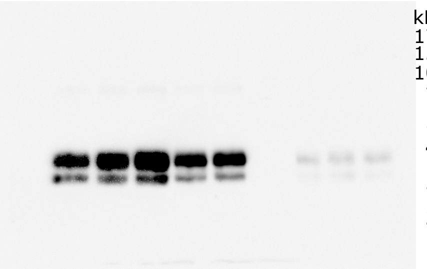

marker

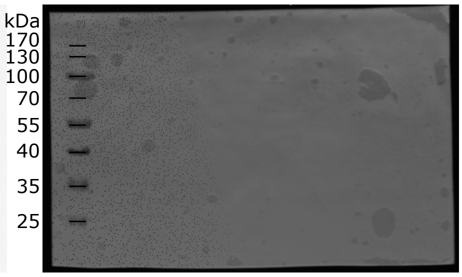

overlayed marker

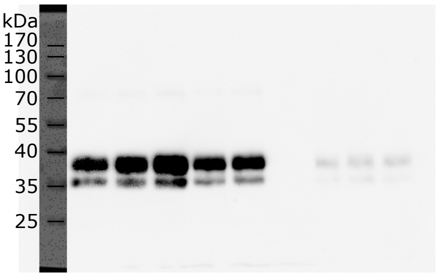

LCK

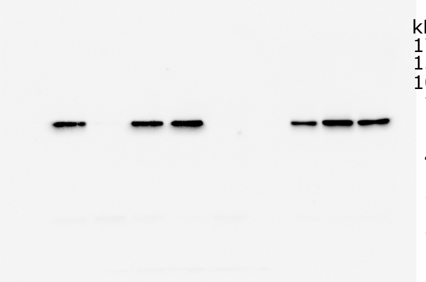

marker

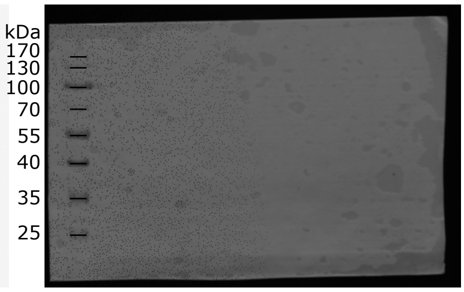

overlayed marker

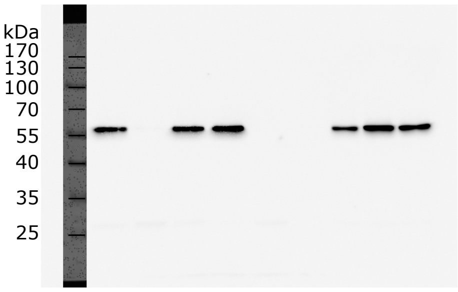

Supplement: Source Data Extended Data Fig. 1 — Uncropped immunoblots. [file 41590_2022_1366_MOESM13_ESM.pdf]
