# Supplementary material for: Unique roles of co-receptor-bound LCK in helper and cytotoxic T cells
Source: Nat Immunol. 2022 Dec 23;24(1):174–85. doi: 10.1038/s41590-022-01366-0 (PMC9810533; doi:10.1038/s41590-022-01366-0)
Supplement: Source Data Extended Data Fig. 2 — Uncropped immunoblots. [file 41590_2022_1366_MOESM15_ESM.pdf]

**ExtData2a**

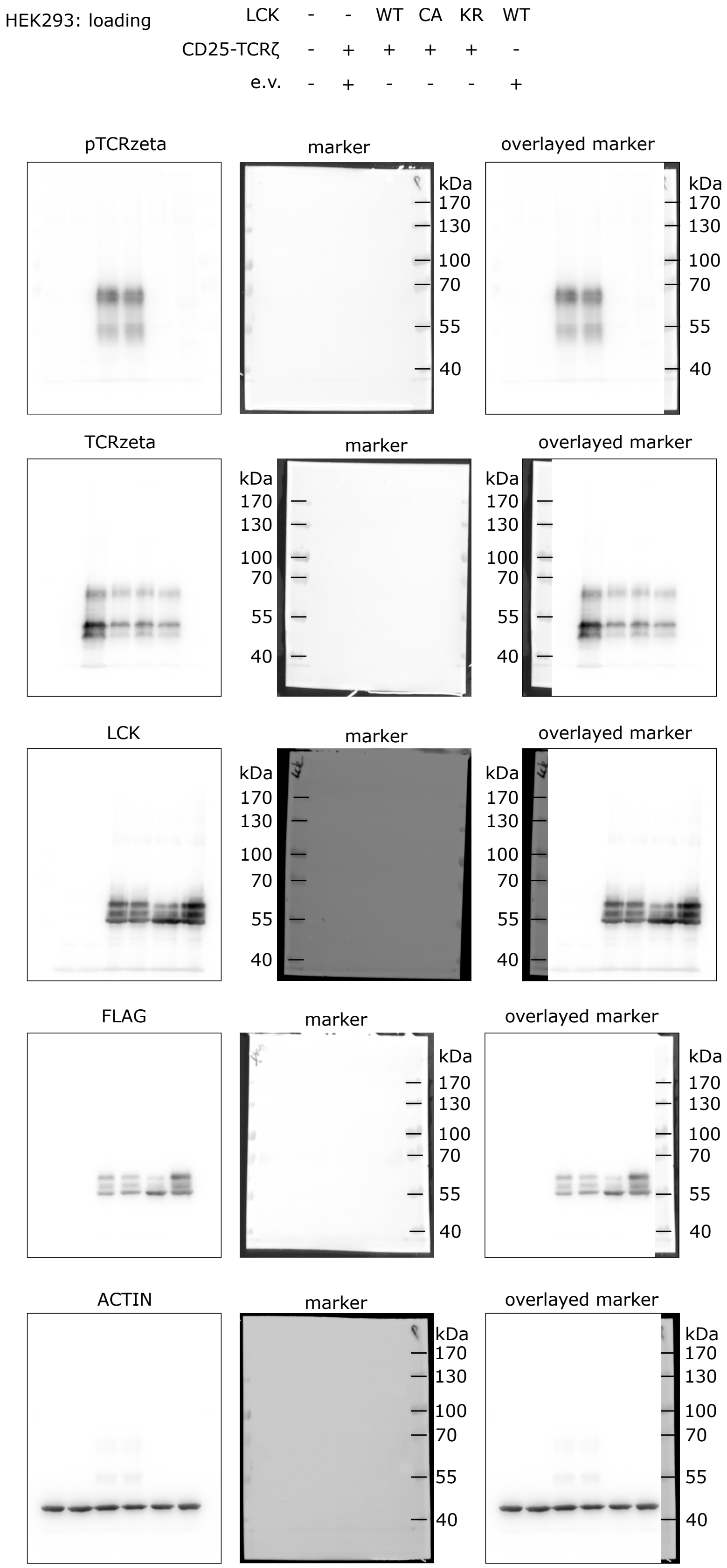
HEK293: loading
LCK - - WT CA KR WT
CD25-TCRζ - + + + + -
e.v. - + - - - +
pTCRzeta
marker
overlayed marker
kDa
170
130
100
70
55
40
TCRzeta
LCK
FLAG
ACTIN

**ExtData2b**

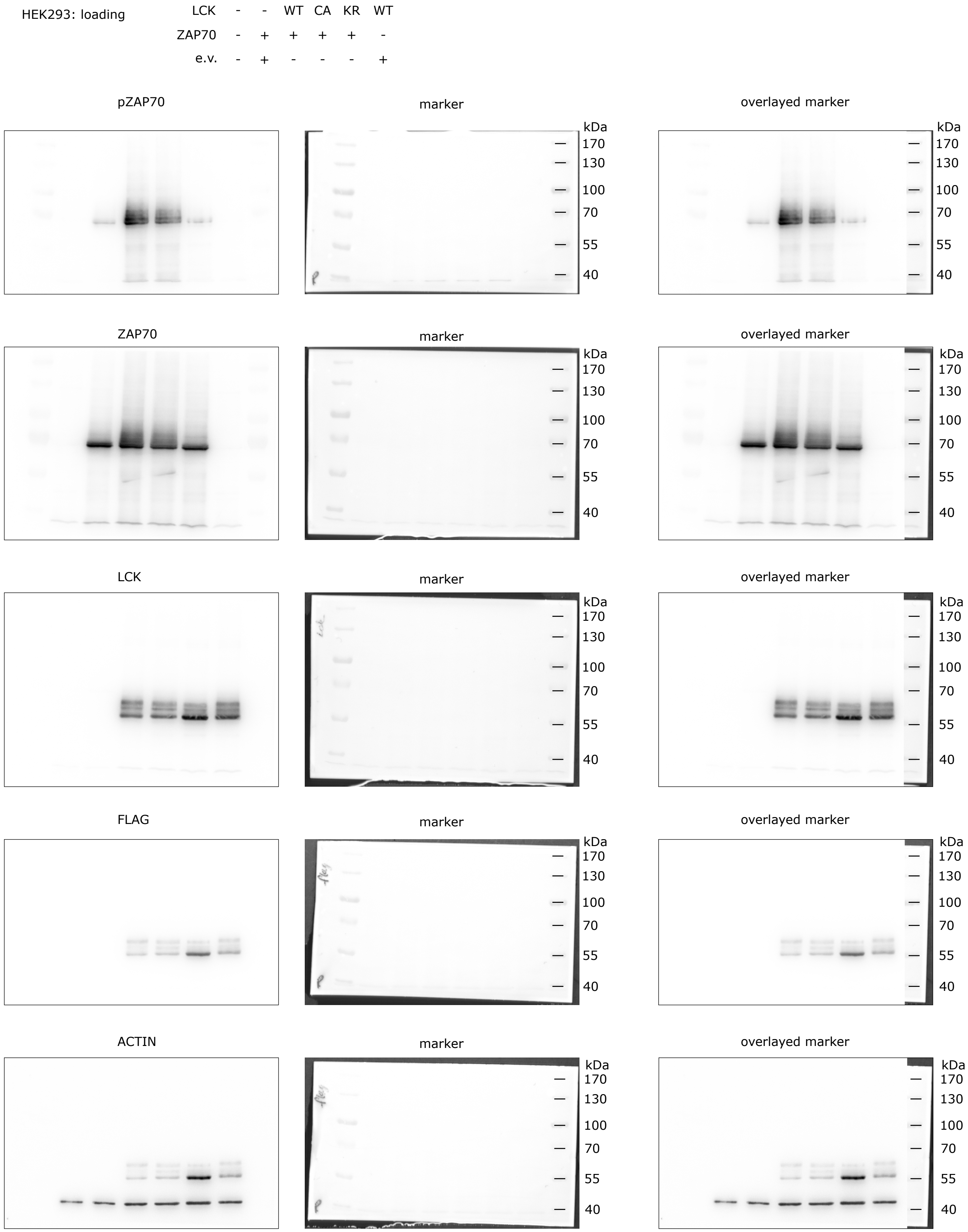
HEK293: loading
LCK - - WT CA KR WT
ZAP70 - + + + + -
e.v. - + - - - +
pZAP70
marker
overlayed marker
kDa
170
130
100
70
55
40
ZAP70
LCK
FLAG
ACTIN

**ExtData2c**

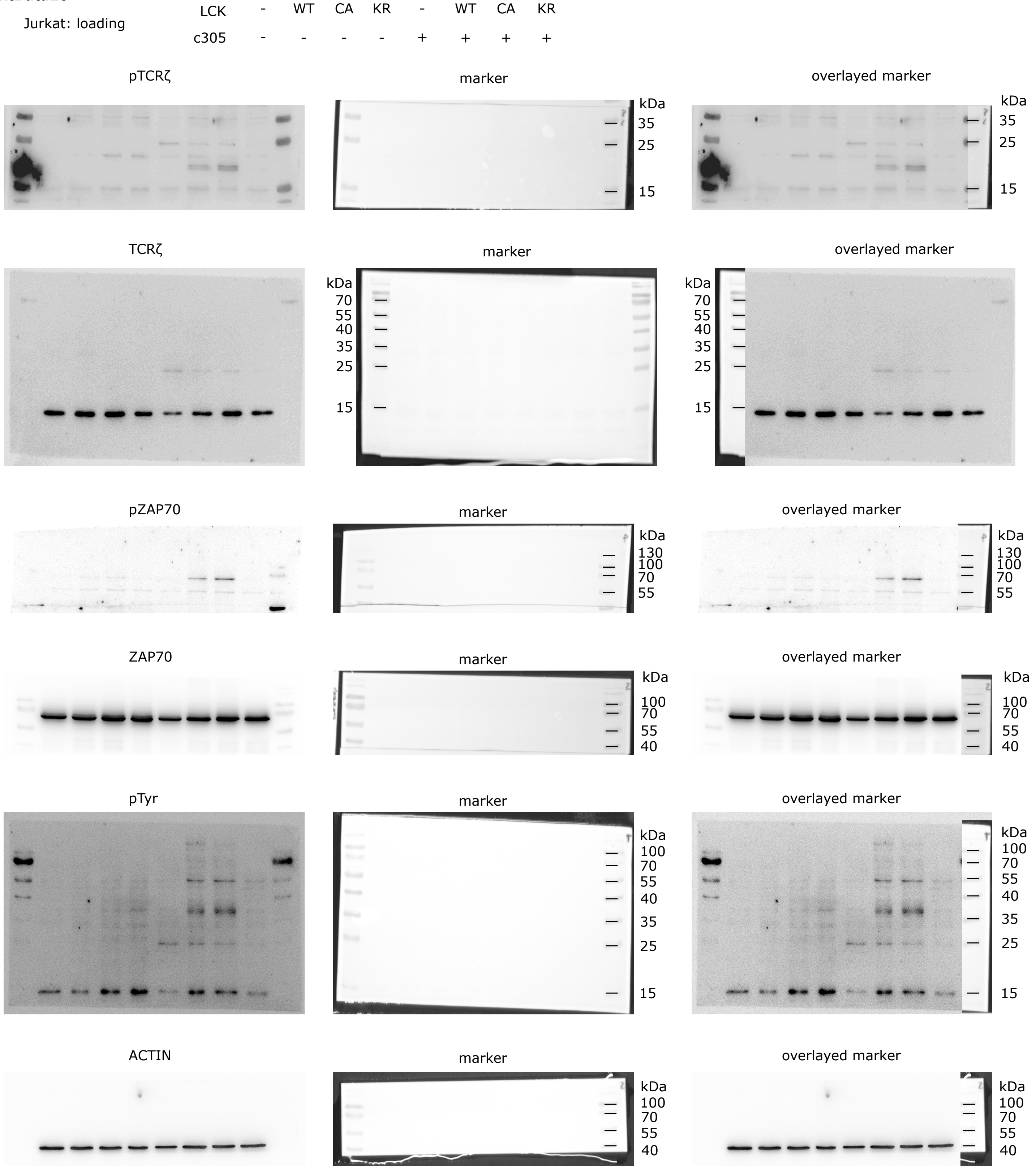

Jurkat: loading
LCK - WT CA KR - WT CA KR
c305 - - - - + + + +
pTCRζ
marker
overlayed marker
kDa
35
25
15
TCRζ
marker
overlayed marker
kDa
70
55
40
35
25
15
pZAP70
marker
overlayed marker
kDa
130
100
70
55
ZAP70
marker
overlayed marker
kDa
100
70
55
40
pTyr
marker
overlayed marker
kDa
100
70
55
40
35
25
15
ACTIN
marker
overlayed marker
kDa
100
70
55
40
